# Implementation science: Relevance in the real world without sacrificing rigor

**DOI:** 10.1371/journal.pmed.1002288

**Published:** 2017-04-25

**Authors:** Elvin H. Geng, David Peiris, Margaret E. Kruk

**Affiliations:** 1 Division of HIV/AIDS, Infectious Diseases and Global Medicine, Department of Medicine, San Francisco General Hospital, University of California, San Francisco, San Francisco, California, United States of America; 2 The George Institute for Global Health, University of Sydney, Sydney, Australia; 3 Department of Global Health and Population, Harvard T.H. Chan School of Public Health, Boston, Massachusetts, United States of America

## Abstract

Three members of *PLOS Medicine*'s editorial board who are leading researchers in implementation science define the characteristics of high-quality studies and invite their submission to the journal.

The need for implementation science in health is now broadly recognized, and a working understanding of the qualities that make an implementation study “good” is needed more than ever before. As defined by Mittman and Eccles, implementation research “is the scientific study of methods to promote the systematic uptake of research findings and other evidence-based practices into routine practice, and, hence, to improve the quality and effectiveness of health services. It includes the study of influences on healthcare professional and organizational behavior” [1]. The scope of implementation science is broad, ranging from observational studies seeking to characterize and understand evidence-practice gaps, to proof-of-concept studies of efficacy, to large-scale implementation and effectiveness trials of complex interventions. Certainly, if findings in this field are not internally valid (i.e., wrong within the source population), they won’t be of use to anyone. But even if findings are internally valid, to be of value, they must be applicable and useful for implementers (e.g., governments, organizations, health care workers, and communities) in diverse real-world contexts. What kinds of findings in implementation science are most useful? Must a trade-off exist between rigor and relevance? If so, what is the right balance between rigor and applicability in a variety of contexts?

The tension between rigor and relevance across contexts is at the center of two conversations in implementation research. One conversation is among investigators immersed in the traditional scientific principles of rigorous human subject research (e.g., sampling, measurement, and confounding) and who must sometimes be persuaded of the importance of usability, applicability, and, therefore, relevance across varied real-world practice contexts. The second conversation is among implementers and evaluators embedded in real-world programs, settings, and populations. Some from this group must be persuaded that rigorous evaluation is needed and that scientific fundamentals, with accompanying effort and planning, are requisite when implementation research is the goal. Fig 1, adapted from Andersen’s heuristic [2] of the Four Ps, summarizes these two conversations.

**Fig 1:**
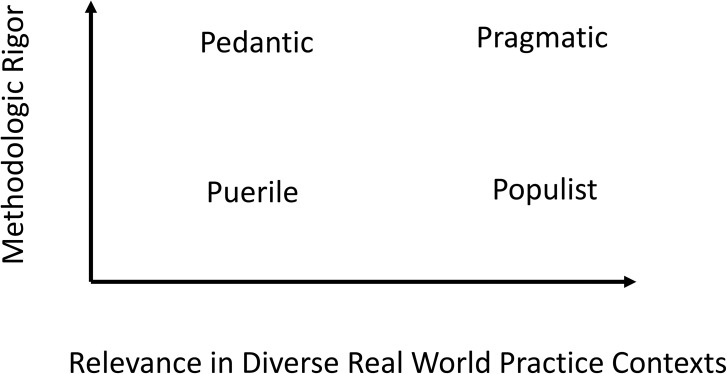
The Four Ps. The vertical axis represents methodological rigor, and the horizontal axis represents relevance in real-world practice. Research low in both dimensions is “puerile.” Rigor without relevance is “pedantic,” while relevance without rigor is “populist.” Implementation research that strives to attain both rigor and relevance is “pragmatic” and the goal. Adapted from Anderson [2].

The first conversation—moving researchers who hail from traditional rigorous clinical investigation toward relevance—is in high gear [3] but faces formidable challenges. Resistance may stem in part from the fact that rules for rigor are well established (and summarized in systems such as Grading of Recommendations Assessment, Development, and Evaluation [GRADE]) [4], while the perspectives for “relevance” (such as Glasgow’s Reach, Effectiveness, Adoption, Implementation, and Maintenance [RE-AIM] framework) [5] are more recent, often require some conjecture, and may seem, to some, flimsy and unscientific. In addition, traditional approaches to rigor in clinical research are sometimes at direct odds with findings that are optimized for relevance. Rigor to enhance internal validity often depends on strict specification of study conditions and participant criteria (e.g., randomized trial of a new medication against placebo). Yet, the more controlled the setting is, the more artificial and less directly informative about impact in real-world settings the participant behaviors are. Even when not in direct conflict, myopic attention to internal validity may sometimes lead to inadvertent neglect of considerations about relevance. For example, use of a randomized trial to evaluate the effect of directly observed antiretroviral therapy for treatment of HIV-infected persons in Africa can yield high-quality scientific evidence by traditional criteria, but selecting a resource-intensive intervention for use in under-resourced health systems may reflect inadequate consideration of fit with real-world practice settings [6].

Fortunately, perspectives from implementation science have highlighted practices that can enhance relevance without compromising internal validity. First, questions most immediately relevant for real-world contexts tend to arise from partnerships between implementers, communities, and researchers [7]. Second, although conceptualization and description of implementation interventions have not always been sufficient to permit replication, use of emerging standards for conceptualizing and reporting of implementation strategies (e.g., Template for Intervention Description and Replication [TIDieR] and Standards for Reporting Implementation Studies [StaRI]) [8–10] can make implementation interventions more transparent. Because studies of implementation strategies are in essence always comparative effectiveness studies, rigorous description of the comparison (the so-called “standard of care”) is just as important as it is for an active intervention. Third, conceptualizing and measuring the mechanisms of effect, and the role of context in those mechanisms, is needed to explain how interventions succeed or fall short in their intended effects, as well as capturing unintended effects [11]. Practical frameworks for process evaluation [12], as well as mixed methods [13] and transdisciplinary approaches, are increasingly common as a means to understand mechanisms. Fourth, implementation outcomes (e.g., reach, adoption, and sustainability) are critical ends in and of themselves in implementation research. Attention to these outcomes sheds light on how interventions were used and how they were adapted (or maladapted) in a particular context [14] and also informs interpretation of effectiveness. Fifth, good implementation science should be cognizant of existing thinking in relevant fields (even if only as a counterpoint) in order to advance knowledge systematically, which can be challenging in an evolving field. Inquiry informed by newer theories from implementation science (e.g., the Consolidated Framework for Implementation Research and the Behavior Change Wheel) or established traditions in the social sciences (e.g., economics and sociology) is best positioned to participate in the ongoing generation of knowledge [15]. Sixth, reporting results of implementation research should be relatively rapid: contextual heterogeneity is true over time as well as across settings in the real world.

Despite an emphasis on relevance across real-world contexts, however, good implementation science is at its core still science. To avoid the pitfalls of post hoc and ad hoc approaches, investigators should prospectively embed evaluation and measurement in the implementation process [16]. To provide valid comparisons, experimental and observational studies both require measurements when one is not (yet) implementing. Study designs like difference in difference, regression discontinuity, the use of instrumental variables, or modern causal methods [17] can strengthen nonexperimental studies in real-world settings. Randomized trials have been criticized as being too slow, expensive, and unable to capture the effects of complex interventions [18] and therefore perhaps not suited for learning about health care delivery [19]. We disagree. Principles for pragmatic trials (e.g., Pragmatic-Explanatory Continuum Indicator Summary 2 [PRECIS-2]) [20] are meant to guide experiments towards results that are applicable to usual care and potentially extricate the strengths of randomization from the artificial demands of traditional trial design. Stepped-wedge cluster randomized trials open the door to strong inferences embedded within real-world programmatic scale-up [21]. Implementation research often uses data (e.g., medical records systems) that are strongly representative of real-world experiences but tend to suffer from missing and misclassified information. Methods to address these flaws such as multiple imputation and bias analysis can enhance the validity of implementation science. In short, the internal validity of scientific claims in implementation science must remain intact for the findings to be useful, whether in a few settings or many.

*PLOS Medicine’s* mission fits with the dual goals of rigor and relevance across contexts in implementation science. The journal seeks to “publish papers on diseases that take the greatest toll on health globally.” Implementation research’s immediate goal is to cross the last mile between efficacious interventions and use in populations for those diseases that have the greatest toll on human health. The journal also seeks to promote “the revolutionary idea of anyone being able to read any article.” [22] Broad access outside of academic settings is particularly important for implementation research, for which the audience is as much implementers (e.g., governments and community-based organizations) as it is researchers. Impactful studies come in diverse designs, and intervention studies should at minimum adequately describe the intervention and the implementation outcomes and carefully address the counterfactual. Such studies can maintain rigor while optimizing relevance and usability across diverse, and sometimes chaotic, real-world contexts and can in turn lead the way to improving health in the real world through this growing field of implementation science. As Academic Editors at *PLOS Medicine* who work in implementation science, we look forward to receiving more research submissions in this growing field.

## Abbreviations

GRADE: Grading of Recommendations Assessment, Development, and Evaluation
PRECIS-2: Pragmatic-Explanatory Continuum Indicator Summary 2
RE-AIM: Reach, Effectiveness, Adoption, Implementation, and Maintenance
StaRI: Standards for Reporting Implementation Studies
TIDieR: Template for Intervention Description and Replication
